# Inferior vena cava anomalous drainage into the left atrium with severe pulmonary arterial hypertension: a case report and review of management strategies

**DOI:** 10.3389/fcvm.2025.1635559

**Published:** 2026-01-12

**Authors:** Lei Yang, Lishi Shao, Feifei Zhou, Yongju Yang, Guifang Sun, Bin Liu

**Affiliations:** Department of Radiology, Yan’an Hospital Affiliated to Kunming Medical University, Kunming, China

**Keywords:** inferior vena cava, left atrium, pulmonary arterial hypertension, case report, anomalous drainage

## Abstract

Anomalous drainage of the inferior vena cava (IVC) into the left atrium is a rare congenital anomaly seldom associated with pulmonary arterial hypertension (PAH). This condition may correlate with atrial septal defect (ASD), pulmonary arteriovenous fistula, and chronic hypoxemia. We present a case of a 34-year-old female admitted with recurrent chest tightness, dyspnea, and palpitations. Comprehensive evaluations, including transthoracic echocardiography, computed tomography angiography, and right heart catheterization, confirmed the diagnosis of IVC anomalous drainage into the left atrium complicated by ASD (about 3 cm), right inferior pulmonary vein anomalous drainage into the inferior vena cava, and severe PAH. Following multidisciplinary consultation, a combined “treat and repair” strategy was adopted, initiating targeted PAH therapy with ambrisentan and sildenafil. At the 3-month follow-up, the patient reported significant symptomatic improvement, resumed daily physical activities without cardiorespiratory discomfort, PASP decreased from 146 mmHg to 127 mmHg and demonstrated enhanced functional capacity. This case highlights the diagnostic challenges and therapeutic considerations for IVC anomalies associated with PH, contributing novel insights to the management of complex congenital cardiovascular disorders.

## Introduction

Inferior vena cava (IVC) anomalous drainage to the left atrium is a rare congenital cardiovascular anomaly, accounting for approximately 0.6% of congenital heart diseases (1). This condition is often associated with atrial septal defects (ASD) and anomalous pulmonary venous drainage (2). However, there are very few reports in the literature regarding IVC anomalous drainage combined with pulmonary arterial hypertension (PAH). As such, the natural clinical course, clinical manifestations, diagnosis, and treatment options for this condition remain underexplored. In contrast to other reported cases, surgical repair is contraindicated in this particular disease, and medical treatment is considered an effective and reasonable approach. We report a rare case of inferior vena cava ectopic drainage into the left atrium accompanied by pulmonary hypertension, as well as a treatment method that differs from those described in other literature.

## Case presentation

A 34-year-old female presented with a history of repeated chest tightness, shortness of breath, and palpitations following a COVID-19 infection two years ago, one year later, the condition worsened, and above symptoms cloud occur frequently with even mild physical activity. Upon seeking treatment at the local county-level hospital, an echocardiogram indicated heart disease, which was not addressed. Subsequently, as symptoms worsened, the patient came to our hospital for care. She has two previous pregnancies. On physical examination, her heart rate and blood pressure were within normal limits, and clubbing of the fingers was observed. Percussion revealed rightward cardiac enlargement, and auscultation revealed a grade 3/6 systolic murmur at the second intercostal space along the left sternal border and a grade 3/6 systolic murmur at the fourth to fifth intercostal spaces along the left sternal border. On admission, her oxygen saturation (SaO₂) was 79%, and her body mass index (BMI) was 18, indicating underweight. Laboratory tests revealed elevated hemoglobin (176 g/L; normal range: 115–150) and NT-proBNP (5,113 ng/L; normal range: ≤450). The electrocardiogram showed ST-T changes, left ventricular hypertrophy, and complete right bundle branch block (Table 1).

**Table 1:** Changes in blood gas analysis during hospitalization.

Parameter	Day 1 (29th Nov)	Day 5 (2nd Dec)	Day 6 (3rd Dec)
PO_2_ (mmHg)	74.5	72.4	76.2
PCO_2_ (mmHg)	29.5	31.8	46.4
SO_2_ (%)	93.2	94.4	94.2
HCO_3_^−^ (mmol/L)	19.0	18.6	24.1
SBC (mmol/L)	21.0	20.5	21.3
SBE (mmol/L)	−4.6	−4.8	−0.9
ABE (mmol/L)	−3.2	−4.6	−1.9
PH (mmol/L)	7.43	7.39	7.34

**Figure 1:**
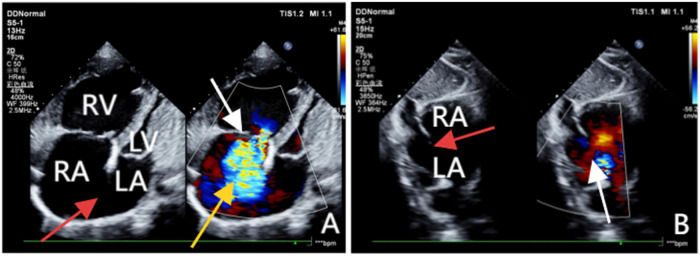
(A**)** Obtained via left lateral decubitus transthoracic echocardiography, comprises two images. The left panel, a two-dimensional view, clearly demonstrates a loss of continuity in the atrial septum and enlargement of the right atrium and ventricle. The right panel, a Doppler ultrasound, highlights the tricuspid valve (white arrow) with a jet of regurgitant blood flow across it (yellow arrow). (B**)** Acquired from the subcostal window with the patient in a supine position, clearly reveals the discontinuity in the atrial septum (red arrow) and a left-to-right shunt across the defect (white arrow).

**Figure 2:**
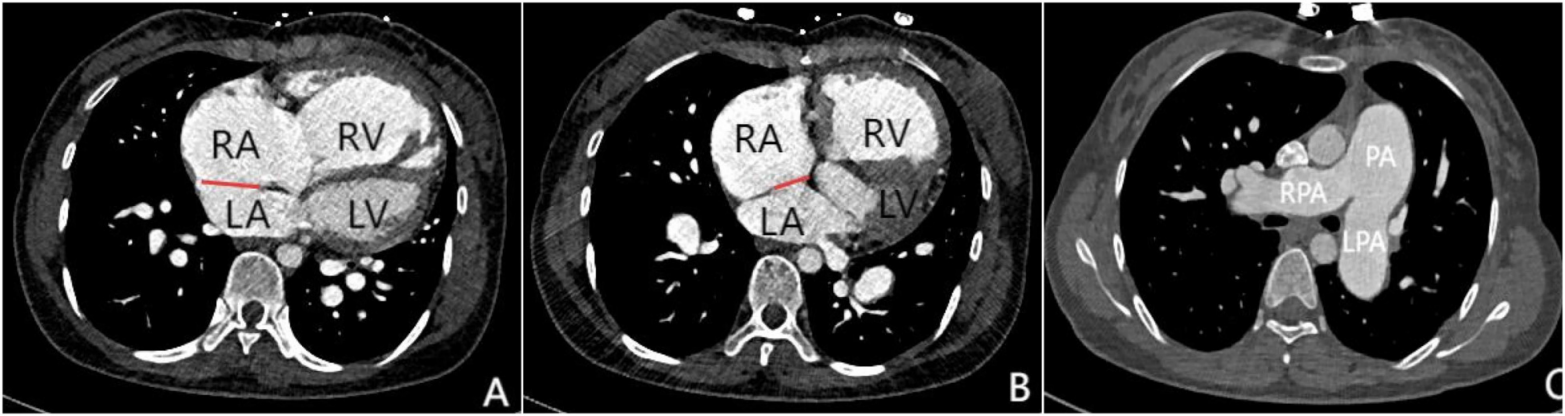
Panels **(A–C)** are all axial images from contrast-enhanced CT scans. Panels **(A and B)** demonstrate atrial septal defects at different locations (indicated by the red lines), measuring 3.1 cm and 1.8 cm in size respectively. Simultaneously, significant enlargement of the right atrium and right ventricle can be observed. Panel **(C)** shows marked dilation of the pulmonary trunk and both left and right pulmonary arteries, which is consistent with signs of pulmonary hypertension.

**Figure 3:**
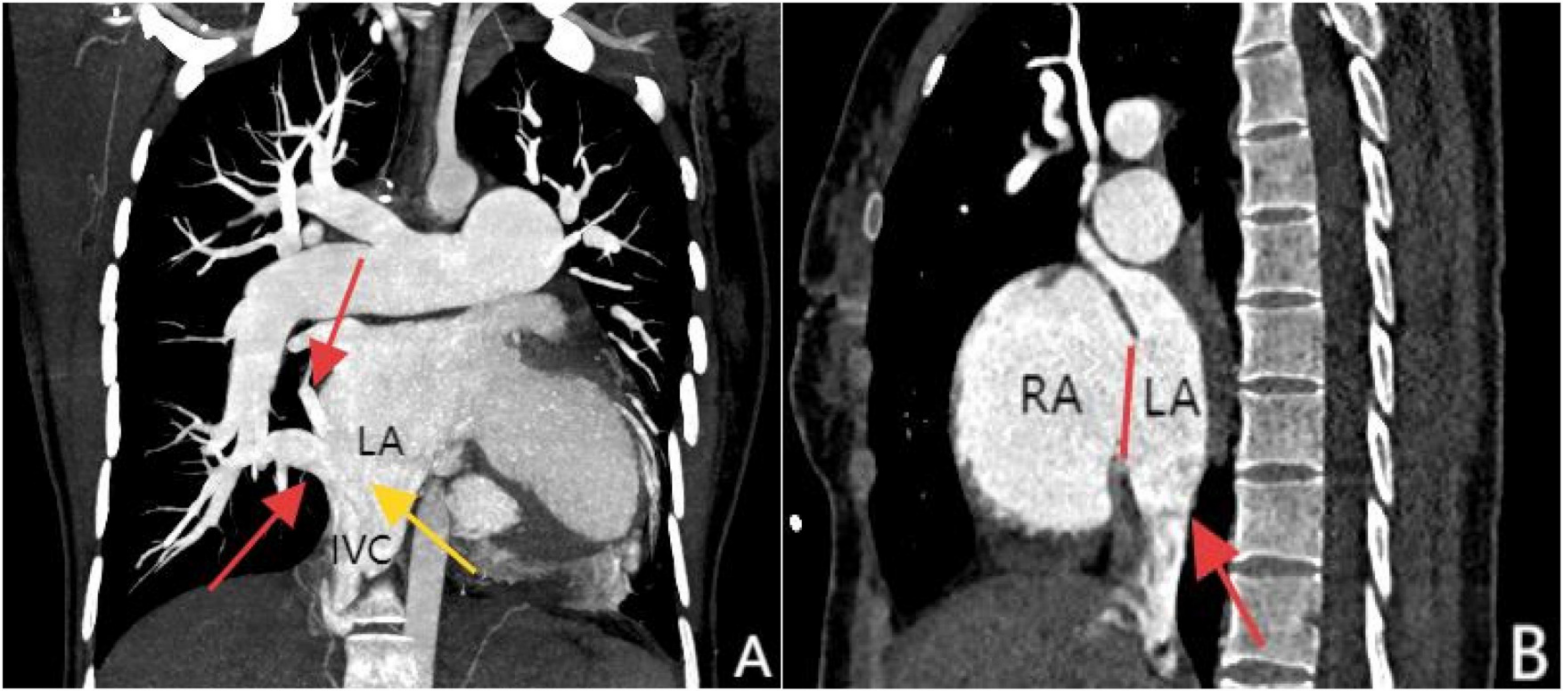
Panel **(A)**, coronal MIP reconstruction, shows that the branches of the right inferior pulmonary vein—specifically the superior segment and basal segments (indicated by the red arrows)—drain into the inferior vena cava at its junction with the left atrium, while the inferior vena cava (marked by the yellow arrow) shows anomalous drainage into the left atrium. Panel **(B)**, contrast-enhanced sagittal view, clearly demonstrates the drainage of the inferior vena cava into the left atrium (red arrow) and an atrial septal defect between the left and right atria (marked by the red line).

**Figure 4:**
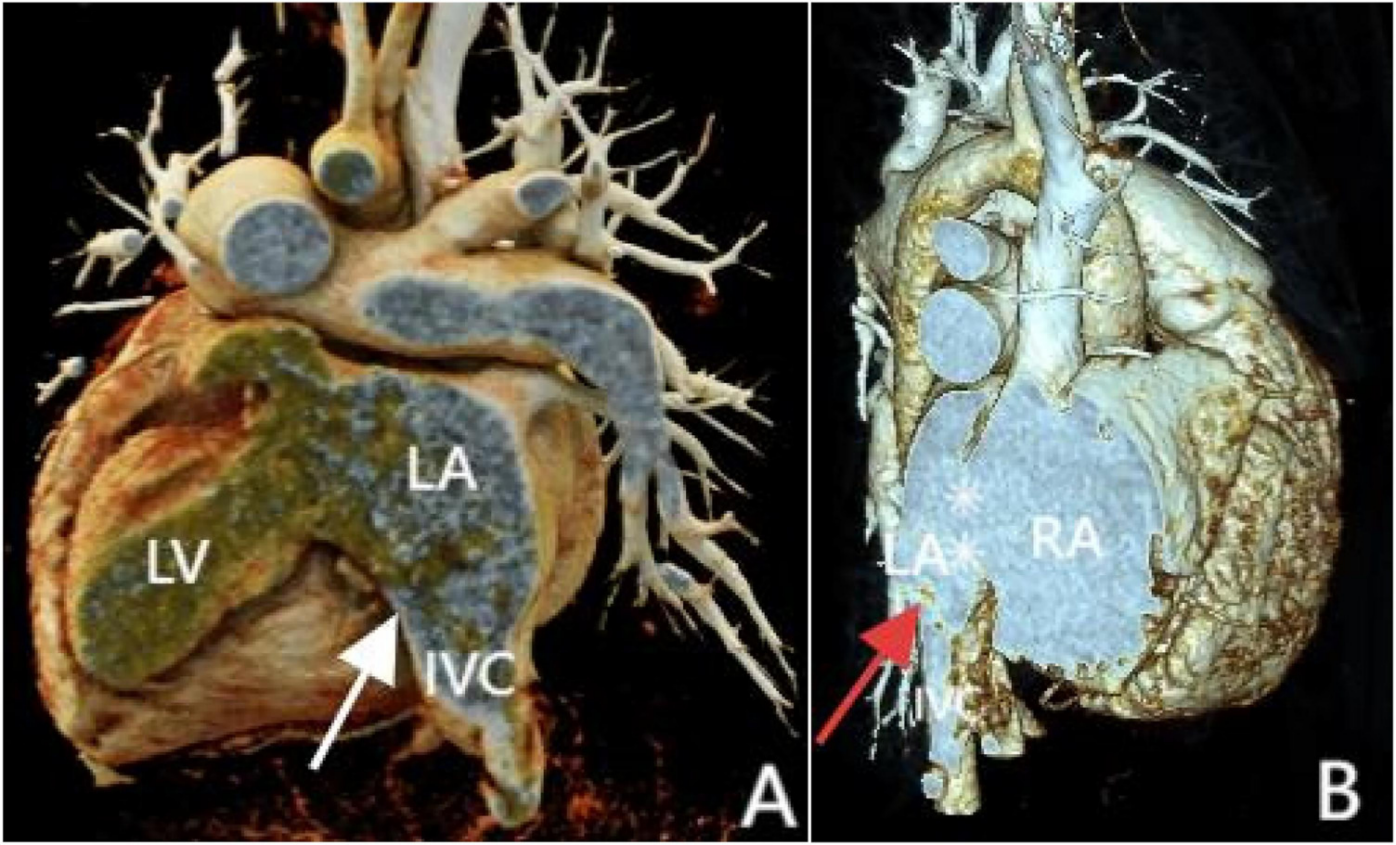
3D reconstruction shows inferior vena cava anomalous drainage into the left atrium (**A and B**, at the white and red arrow locations) and atrial septal defect (**B**, at the white asterisk). Panel **(A)** shows the posterior view, and Panel **(B)** shows the front view.

Transthoracic echocardiography (TTE) revealed an atrial septal defect (ASD) with a 3 cm opening (Figure 1), along with partial anomalous pulmonary venous drainage. Pulmonary artery systolic pressure (PASP) was 147 mmHg by measuring the tricuspid regurgitation spectrum using continuous Doppler., and severe tricuspid regurgitation, the inner diameter of the main pulmonary artery widened slightly, about 3 cm, left ventricular ejection fraction(LVEF) 69% were noted. Coronary CT angiography (CTA) confirmed these abnormalities and revealed a multi-fenestrated ASD (measuring 2.8 × 3.1 cm and 1.8 × 1.9 cm), the dorsal and basal segmental branches of the right inferior pulmonary vein drain abnormally into the inferior vena cava at its junction with the left atrium. Chest and abdominal CT angiography (CTA) further confirmed the IVC anomaly draining into the left atrium (Figures 2, 3). Right heart catheterization confirmed severe pulmonary hypertension, revealing a pulmonary artery pressure of 117/40 mmHg (mean 60 mmHg). While a full set of hemodynamic measurements including cardiac output was not obtained during the procedure, the documented pulmonary artery pressures were sufficient to establish the diagnosis of severe PAH and guide the initial therapeutic strategy (Figure 4).

Upon admission, the patient was immediately given nasal oxygen therapy and oral potassium chloride for potassium supplementation, while taking spironolactone and furosemide for diuresis, and metoprolol sustained-release tablets to improve cardiac function as part of the basic treatment. Based on the recommendations from the 2020 ESC Guidelines for Adult Congenital Heart Disease regarding the need for preoperative reduction of pulmonary vascular resistance in patients with pulmonary hypertension, after discussion by a multidisciplinary team, the treatment decision for this patient is as follows: prior to surgical correction, bridging therapy with targeted drugs is to be used first, specifically the combination of the endothelin receptor antagonist ambrisentan and the phosphodiesterase-5 inhibitor sildenafil. The patient will undergo a follow-up after 3 months treatment to reassess pulmonary artery pressure and determine the potential for surgical intervention.

The patient returned for follow-up after 3 months of targeted medical therapy. Echocardiography revealed a reduction in pulmonary arterial systolic pressure (PASP) from 146 mmHg at baseline to 127 mmHg, with a preserved left ventricular ejection fraction (LVEF) of 67%. Laboratory studies showed a significant decrease in N-terminal pro-brain natriuretic peptide (NT-proBNP) to 2,798 ng/L, compared to the pre-treatment level of 5,113 ng/L. During a subsequent telephone follow-up, the patient reported notable alleviation of previous symptoms such as chest tightness and shortness of breath, with no significant exercise intolerance during routine daily activities.

Although the underlying congenital anomaly—anomalous drainage of the inferior vena cava into the left atrium—remains unchanged on echocardiography, the combination of hemodynamic improvement (reduced PASP), favorable biomarker trend (decline in NT-proBNP), and symptomatic relief collectively indicate enhanced cardiac function and support the therapeutic efficacy of the current pharmacological regimen.

## Discussion

Diagnosing IVC anomalous drainage to the left atrium is challenging due to its nonspecific clinical presentation. As a result, many patients are not diagnosed until adulthood. In this case, despite an SaO₂ of only 79%, the patient showed no significant cyanosis except for clubbing. This atypical clinical presentation makes early diagnosis particularly challenging, especially in the absence of multimodal imaging examinations. TTE, as the first-line screening tool, did not detect the IVC anomaly. However, coronary CTA revealed an ASD and other associated abnormalities. It was not until chest and abdominal CTA that the IVC anomaly draining into the left atrium was identified. This process highlights the importance of multimodal imaging in the diagnosis of complex cardiovascular abnormalities. In early reported cases, no abnormalities were detected in cardiac or pulmonary screenings using x-ray examinations until postmortem autopsy revealed anomalous drainage of the inferior vena cava (IVC) into the left atrium (10). Although later studies introduced diagnostic tools such as Doppler echocardiography, most cases were ultimately confirmed through invasive angiography and catheterization. While angiography directly visualizes IVC anomalous drainage, its invasive nature and associated procedural risks remain a concern for patients and their families. With advancements in diagnostic imaging, clinicians now increasingly combine ultrasonography, CT, MRI, and three-dimensional reconstruction for comprehensive diagnosis of this condition. For example, Atakan et al. (3) utilized transthoracic echocardiography (TTE) and CT angiography (CTA), while Surendrasingh et al. employed MRI to confirm the diagnosis. Thus, a multi-modal imaging approach, incorporating echocardiography, angiography, MRI, or CT, is often necessary to firmly establish the diagnosis, because single examination methods can easily lead to missed or misdiagnosed cases, whereas the combined use of various imaging technologies can significantly improve the accuracy of diagnosis (4).

The systemic sinus venosus and the primary part of the right atrium are separated during the early stages of embryologic development by the right and left venous valves. The crista terminalis, Eustachian, and Thebesian valves are typically all that remain of the right sinus venosus valve once it atrophies. The IVC will drain into the left atrium if the right sinus venosus valve does not regress and connect with the superior section of the septum secundum (5). Elevated left atrial pressure and volume, along with reduced left atrial compliance, lead to passive transmission of pressure into the pulmonary vasculature. This results in structural remodeling of the vessels, characterized by intimal fibrosis and hypertrophy, which impairs vasodilatory capacity and promotes pulmonary vasoconstriction, thereby increasing pulmonary vascular resistance (PVR) (6). Concurrently, elevated pulmonary arterial pressure reduces nitric oxide (NO) production, shifts the prostaglandin-thromboxane balance toward thromboxane A_2_ (TXA_2_), and upregulates endothelin synthesis. These changes collectively induce vasoconstriction, platelet aggregation, and smooth muscle cell proliferation (7), which together drive the progression of pulmonary hypertension.

The anomalous drainage of the inferior vena cava (IVC) into the left atrium was first reported in 1955 by Gardner, who described it as an incidental autopsy finding (11). Subsequent literature gradually documented this rare anomaly, though primarily in the form of isolated case reports (12). In 1965, Miller et al. successfully performed surgical correction for anomalous drainage of the inferior vena cava (IVC) (13). Since then, almost all literature has primarily adopted surgical treatment as the standard approach. However, none of the studies I reviewed discussed whether surgical intervention remains feasible for cases of IVC anomalous drainage complicated by pulmonary hypertension (PAH). Contrary to the previously reported treatment strategies, we argue that severe PAH is precisely a contraindication for surgery. For such patients, we recommend a “treat-and-repair” strategy: first using targeted medications the endothelin receptor antagonist （such as ambrisentan）,and the phosphodiesterase-5 (PDE-5,such as sildenafil), followed by reevaluation of surgical options through right heart catheterization and angiography to assess right heart function. Surgical procedures like atrial septal defect closure or IVC drainage correction should only be performed after pulmonary vascular resistance is reduced to below 5 Wood units. This approach has been proven to reduce perioperative mortality in patients with high pulmonary vascular resistance and improve outcomes (14, 15). For this particular case, after multidisciplinary discussion, it was decided to follow guideline recommendations and initiate specific targeted therapy with ambrisentan and sildenafil to lower pulmonary arterial pressure prior to surgical correction. The potential benefits of monotherapy with either sildenafil or ambrisentan in PAH management are well-established. However, studies have shown that combining ambrisentan with sildenafil significantly improves 6-minute walk test results and NT-proBNP levels in PAH patients compared to sildenafil alone (16–18).

In light of the advanced pulmonary vascular remodeling and the absence of pulmonary venous obstruction, the patient's condition is best characterized as Eisenmenger syndrome. The atrial septal defect is no longer a simple shunt but a vital communication in this moment that aids survival by allowing right-to-left shunting, thereby offloading the failing right ventricle. Therefore, surgical intervention to correct the defect would be detrimental, as it could trigger severe right heart failure and a pulmonary hypertensive crisis. Our management has thus correctly focused on a “treat-only” strategy with pulmonary vasodilators, the efficacy of which has been confirmed by follow-up.

Atrial septal defect (ASD) is a common cause leading to pulmonary arterial hypertension (PAH). Studies have shown that approximately 35% of patients with ASD will eventually develop pulmonary arterial hypertension if surgery is not performed in a timely manner (8). In this case, the patient not only had ASD but also presented with anomalous drainage of the inferior vena cava and the dorsal and basal segmental branches of the right inferior pulmonary vein drain abnormally into the inferior vena cava, which together caused severe pulmonary arterial hypertension. Notably, pregnancy and COVID-19 infection maybe are known risk factors for exacerbating PAH. Pregnancy and delivery significantly increase cardiovascular burden, particularly for patients with congenital heart disease. The patient in this case has a history of two pregnancies, which may further exacerbate her pulmonary arterial hypertension. Studies have shown that pregnant women with PAH are at a higher risk of mortality and complications, especially during the late stages of pregnancy and perioperatively. Additionally, acute COVID-19 infection worsens hypoxemia and increases the risk of thrombosis in PAH patients, leading to higher complication rates and mortality in congenital heart disease (CHD) populations. The COVID-19 virus may exacerbate myocardial dysfunction through overwhelming immune-inflammatory responses, direct viral invasion of myocardial cells, or severe hypoxia-induced myocardial ischemia. These mechanisms may further complicate the condition of CHD patients with preexisting myocardial dysfunction or pulmonary vascular disease (9, 10). Therefore, individualized care is essential for CHD patients who contract COVID-19 and pregnancy.

## Conclusion

There are few reports in the literature regarding IVC anomalous drainage to the left atrium in conjunction with ASD and anomalous pulmonary venous drainage. This case, through multimodal imaging diagnostics and a “treat-and-repair” strategy primarily targeting pulmonary arterial hypertension (PAH) with pharmacological therapy, provides valuable insights for the diagnosis and clinical management of similar patients. In patients with prolonged undiagnosed or recurrent cyanosis, a high degree of vigilance is required for the possibility of right-to-left shunting and abnormal venous drainage. In addition to anatomical diagnosis, comprehensive management must also include formal prognostic assessment using established risk parameters to guide treatment intensity and patient counseling. Furthermore, considering that pulmonary arterial hypertension (PAH) may have a genetic basis, referral for genetic counseling and testing is a key component of a complete evaluation. Finally, pregnancy and COVID-19 infection are factors that may precipitate clinical deterioration, so individualized management and treatment are necessary for these patients.

## Limitation

This study is limited by its short-term follow-up period of only 3 months. Longer-term data are necessary to confirm the durability of the treatment response and to monitor for potential late-onset adverse events. Inaddition, lack of a complete hemodynamic assessment also is a limitation in this report, when evaluate similar complex cases should aim for a full set of catheterization data, including pulmonary vascular resistance calculation, to better inform long-term management and prognosis in the future.

## Data Availability

The original contributions presented in the study are included in the article/Supplementary Material, further inquiries can be directed to the corresponding authors.

## References

[1] Wang X Zhou M Wang L Han L Li P Nie F Abnormal drainage of inferior vena cava to left atrium combined with atrial septal defect: a case report. Int J Surg Case Rep. (2022) 96:107384. 10.1016/j.ijscr.2022.107384 35803100 PMC9284046

[2] Aliter H El-Haddad A Gallo R Al-Halees Z. Atrial septal defect with drainage of the inferior vena cava into the left atrium. Eur J Cardiothorac Surg. (2011) 40:1256–7. 10.1016/j.ejcts.2011.02.011 21420312

[3] Asotra S Kumar R Dhaulta P Verma M. Anomalous drainage of inferior vena cava into left atrium associated with ostium secundum atrial septal defect: a case report. Eur Heart J Case Rep. (2024) 8:ytae293. 10.1093/ehjcr/ytae293 38983455 PMC11232694

[4] Prakash A Pandey P Khonde IP Goyal A. Inferior vena cava draining into the left atrial cavity due to atrial septal defect: two atypical presentations. Cureus. (2023) 15(5):e39159. 10.7759/cureus.39159 37332418 PMC10275708

[5] Al-Omary MS Sugito S Boyle AJ Sverdlov AL Collins NJ. Pulmonary hypertension due to left heart disease: diagnosis, pathophysiology, and therapy. Hypertension. (2020) 75(6):1397–408. 10.1161/HYPERTENSIONAHA.119.14330 32336230

[6] Lan NSH Massam BD Kulkarni SS Lang CC. Pulmonary arterial hypertension: pathophysiology and treatment. Diseases. (2018) 6(2):2–38. 10.3390/diseases6020038 PMC6023499 29772649

[7] Engelfriet PM Duffels MG Möller T Boersma E Tijssen JG Thaulow E Pulmonary arterial hypertension in adults born with a heart septal defect: the euro heart survey on adult congenital heart disease. Heart. (2007) 93(6):682–7. 10.1136/hrt.2006.098848 17164490 PMC1955187

[8] Chivers S Cleary A Knowles R Babu-Narayan SV Simpson JM Nashat H COVID-19 in congenital heart disease (COaCHeD) study. Open Heart. (2023) 10(2):e002356. 10.1136/openhrt-2023-002356 37460271 PMC10357297

[9] Madjid M Safavi-Naeini P Solomon SD Vardeny O. Potential effects of coronaviruses on the cardiovascular system: a review. JAMA Cardiol. (2020) 5:831–40. 10.1001/jamacardio.2020.1286 32219363

[10] Gardner DL Cole L. Long survival with inferior vena cava draining into left atrium. Brit Heart J. (1955) 17:93–101. 10.1136/hrt.17.1.93 13230352 PMC503890

[11] Atalay A Ecevit AN. The anomalous drainage of the inferior vena cava into the left atrium. Turkish J Pediatr. (2022) 64(5):932–4. 10.24953/turkjped.2021.4320 36305445

[12] Chhabada S Khanna S. Anomalous drainage of inferior vena cava into the left atrium. Anesthesiology. (2018) 129:532–4. 10.1097/ALN.0000000000002169 29498946

[13] Miller GA Ongley PA Rastelli GC Kirklin JW. Surgical correction of total anomalous systemic venous connection: report of case. Mayo Clin Proc. (1965) 40:532–7. PMID: 14346188 14346188

[14] Takaya Y Akagi T Sakamoto I Kanazawa H Nakazawa G Murakami T Efficacy of treat-and-repair strategy for atrial septal defect with pulmonary arterial hypertension. HEART. (2022) 108(5):382–7. 10.1136/heartjnl-2021-319096 34415851 PMC8862039

[15] Baumgartner H De Backer J Babu-Narayan SV Budts W Chessa M Diller GP 2020 ESC guidelines for the management of adult congenital heart disease. Eur Heart J. (2021) 42:563–645. 10.1093/eurheartj/ehaa554 32860028

[16] Mohammed S Vijayvergiya R Malhotra S Rohit MK. A randomized, double-blind, placebo-controlled study to evaluate sildenafil, ambrisentan combination therapy in pulmonary hypertension, particularly of Eisenmenger syndrome. Indian Heart J. (2021) 73:S362–7. 10.1016/j.ihj.2021.07.007 PMC8514406 34627582

[17] Iversen K Jensen AS Jensen TV Vejlstrup NG Søndergaard L. Combination therapy with bosentan and sildenafil in Eisenmenger syndrome: a randomized, placebo-controlled, double-blinded trial. Eur Heart J. (2010) 31:1124e1131. 10.1093/eurheartj/ehq011 20202971

[18] Hassoun PM Zamanian RT Damico R Lechtzin N Khair R Kolb TM Ambrisentan and tadalafil up-front combination therapy in scleroderma-associated pulmonary arterial hypertension. Am J Respir Crit Care Med. (2015) 192:1102e1110. 10.1164/rccm.201507-1398OC 26360334 PMC4642204

